# Discovery of Bat Coronaviruses through Surveillance and Probe Capture-Based Next-Generation Sequencing

**DOI:** 10.1128/mSphere.00807-19

**Published:** 2020-01-29

**Authors:** Bei Li, Hao-Rui Si, Yan Zhu, Xing-Lou Yang, Danielle E. Anderson, Zheng-Li Shi, Lin-Fa Wang, Peng Zhou

**Affiliations:** a CAS Key Laboratory of Special Pathogens, Wuhan Institute of Virology, Center for Biosafety Mega-Science, Chinese Academy of Sciences, Wuhan, China; b University of Chinese Academy of Sciences, Beijing, China; c Programme in Emerging Infectious Diseases, Duke-NUS Medical School, Singapore, Singapore; University of Maryland, College Park

**Keywords:** bat, coronavirus, genome, enrichment, next-generation sequencing

## Abstract

Coronaviruses (CoVs) of bat origin have caused two pandemics in this century. Severe acute respiratory syndrome (SARS)-CoV and Middle East respiratory syndrome (MERS)-CoV both originated from bats, and it is highly likely that bat coronaviruses will cause future outbreaks. Active surveillance is both urgent and essential to predict and mitigate the emergence of these viruses in humans. Next-generation sequencing (NGS) is currently the preferred methodology for virus discovery to ensure unbiased sequencing of bat CoVs, considering their high genetic diversity. However, unbiased NGS is an expensive methodology and is prone to missing low-abundance CoV sequences due to the high background level of nonviral sequences present in surveillance field samples. Here, we employ a capture-based NGS approach using baits targeting most of the CoV species. Using this technology, we effectively reduced sequencing costs by increasing the sensitivity of detection. We discovered nine full genomes of bat CoVs in this study and revealed great genetic diversity for eight of them.

**IMPORTANCE** Active surveillance is both urgent and essential to predict and mitigate the emergence of bat-origin CoV in humans and livestock. However, great genetic diversity increases the chance of homologous recombination among CoVs. Performing targeted PCR, a common practice for many surveillance studies, would not reflect this diversity. NGS, on the other hand, is an expensive methodology and is prone to missing low-abundance CoV sequences. Here, we employ a capture-based NGS approach using baits targeting all CoVs. Our work demonstrates that targeted, cost-effective, large-scale, genome-level surveillance of bat CoVs is now highly feasible.

## Abstract (precis)

Active surveillance is both urgent and essential to predict and mitigate the emergence of bat-origin CoV in humans and livestock. However, great genetic diversity increases the chance of homologous recombination among CoVs. Performing targeted PCR, a common practice for many surveillance studies, would not reflect this diversity. NGS, on the other hand, is an expensive methodology and is prone to missing low-abundance CoV sequences. Here, we employ a capture-based NGS approach using baits targeting all CoVs. Our work demonstrates that targeted, cost-effective, large-scale, genome-level surveillance of bat CoVs is now highly feasible.

## INTRODUCTION

Coronaviruses (CoVs) have the largest nonsegmented genomes among all RNA viruses, reaching up to 30 kb in length. The large genomes enhance plasticity, thereby allowing modification by mutations and recombination, which in turn leads to greater genetic diversity and high chances of cross-species transmission (1, 2). The major reason for this phenomenon may be the numerous subgenomic RNAs generated during viral replication, which increase the chance of homologous recombination among closely related genes from different lineages of CoVs or other viruses (3, 4). As a result, CoV taxonomy is constantly changing. Currently, there are four genera (*Alpha-*, *Beta-*, *Gamma-*, and *Deltacoronavirus*) consisting of 38 unique species in the CoV subfamily *Orthocoronavirinae*, and the number is still increasing (5). Open reading frame 1b (ORF1b) is the gene used for classification, but viruses in the same species may show great diversity in regions outside ORF1b, confounding the designation (6). Bat CoVs classed as the same species can differ significantly in terms of receptor usage or virus-host interaction, as observed in bat severe acute respiratory syndrome (SARS)-related CoVs (SARSr-CoVs) (7). This difference would not be reflected by performing targeted PCR on short genomic fragments of ORF1b, currently a common practice for many surveillance studies (8).

Over the past 20 years, two pandemics, SARS and Middle East respiratory syndrome (MERS), have been attributed to CoVs (9, 10). The outbreak in 2018 of swine acute diarrhea syndrome (SADS), another bat CoV, is a timely reminder that CoVs will continue to emerge and cause new outbreaks in the future (11). All three disease agents can be traced back to bats, animals known to harbor other deadly viruses, including Ebola virus, Marburg virus, Nipah virus, and Hendra virus (12). Bat CoVs are highly prevalent around the world and also show great genetic diversity, making up almost 60% of all known *Alpha*- and *Betacoronavirus* species. It is generally believed that some of these bat CoVs have the potential to spill over into humans and other mammalian species, causing another SARS-like pandemic (13). While predicting the potential spillover and emergence of a novel bat coronavirus is difficult, active surveillance is a valuable monitoring mechanism. Surveillance programs have been designed to aid viral discovery in wildlife reservoir hosts to mitigate infection and emergence in the human population. These programs propose to use next-generation sequencing (NGS) and other approaches to ensure unbiased evolutionary analysis of bat CoVs that takes into consideration their high genetic diversity (14). In order to be effective, these types of surveillance programs rely upon processing of samples in a high-throughput manner and require the compilation of whole-genome sequences. Although NGS enables unbiased pathogen discovery, implementation of this methodology for virus surveillance is costly. Additionally, the inherent lack of sensitivity with an unbiased approach increases the burden of data analysis and decreases the chance of detection in field samples with low viral loads.

Strategies to improve the efficiency of NGS have been explored, including subtraction of host genetic material or enrichment of viral nucleic acid through positive selection using a capture-based system, where the latter was proven more cost-effective (15–17). Virus enrichment NGS has been successfully used for various viral families, and the most common protocols rely on predesigned viral probes that share more than 60% homology with the target virus sequence (15–17). In this study, we utilized an enrichment NGS approach with predesigned probes targeting most of the CoV species (18). Our aim was to strategically perform bat CoV surveillance in which high-throughput sample processing for virus discovery would be balanced with cost effectiveness. Ultimately, the aim is to determine the best strategy to mitigate potential virus emergence in the future.

## RESULTS

### Enrichment NGS aids in the detection and characterization of diverse CoVs.

In surveillance studies, detection and characterization are fundamental requirements to fully assess the risk that bat CoVs pose to humans. We aimed to address two main issues encountered during surveillance studies. First, many samples are collected but not all samples harbor viruses. Second, when viruses are detected, the high genetic diversity of CoVs means that full-length genome sequencing is essential to fully characterize viruses. In the context of CoV discovery, it is not time or cost-effective to perform unbiased NGS on all samples. Most data generated from unbiased NGS can be attributed to non-CoV-specific reads. To assess whether samples can be enriched to allow sequencing of only CoV-specific reads, we utilized NGS in conjunction with viral nucleic acid capture specifically targeting most of the known CoVs using a pool of 4,303 unique baits (18). These baits were designed from 90 representative CoV genomes, and *in silico* analysis determined that these baits should target all known CoV species tested here (Table S2 in the supplemental material).

10.1128/mSphere.00807-19.2 TABLE S2 Information on reference genomes and samples used in this study. Bat CoV species are highlighted in blue. A short 440-bp RdRp region was aligned to the reference genome, and percentages of identity are shown. Numbers of expected functional baits were counted by blasting 4,303 CoV baits against genomes retrieved, and >80% homology is acceptable. Viral loads were determined by qPCR targeting the 440-bp RdRp region and are shown as *C_T_* values. Download Table S2, XLSX file, 0.01 MB. Copyright © 2020 Li et al. 2020 Li et al. This content is distributed under the terms of the Creative Commons Attribution 4.0 International license.

**FIG 1:**
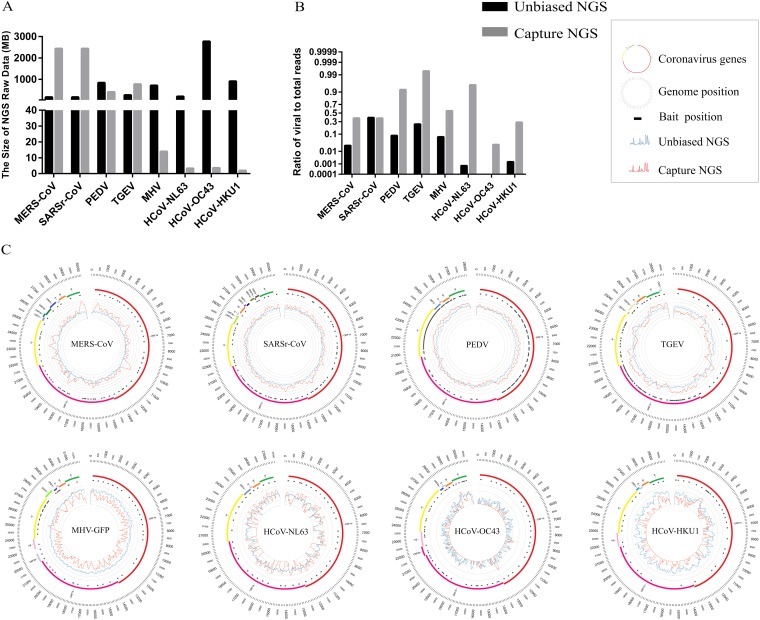
Next-generation sequencing (NGS) using a coronavirus (CoV) enrichment approach. Five cultured viruses (SARSr-CoV, MERS-CoV, PEDV, TGEV, and MHV) and three human clinical samples (HCoV-HKU1, HCoV-OC43, and HCoV-NL63) were used. (A) Amount of NGS data in megabytes (Mb). Data amounts were compared between unbiased and enriched NGS. (B) Ratios of viral to total reads were determined by mapping reads to the respective reference genome using CLC genomics. (C) CoV Circos plots. RNA extracted from cell culture supernatant or human oral swabs was subjected to NGS analysis. Circos plots, from outer to inner circle: CoV genome length (bp), genome annotation, CoV bait positions, read depth from direct NGS (blue lines), and read depth from enrichment NGS (red lines). Scale of read depth is shown as seven thin circular lines and ranges from 0 to 10^6^. Sample details can be found in Table S2 in the supplemental material.

A panel of 5 diverse CoVs (SARSr-CoV, MERS-CoV, porcine epidemic diarrhea virus [PEDV], transmissible gastroenteritis coronavirus [TGEV], and mouse hepatitis virus [MHV]) were amplified in cell culture, and RNA was extracted from the supernatants. Additionally, to test the robustness of the assay, RNA was extracted from 3 clinical samples (oral swabs from humans infected with human CoV OC43 [HCoV-OC43], HCoV-HKU1, and HCoV-NL63). NGS libraries were constructed and either directly sequenced or subjected to enrichment prior to sequencing. The 17 captured samples were made into two pools (8 or 9 per pool) for sequencing. The total amount of data obtained was variable across samples, but in swabs, unbiased NGS consistently produced more data (Fig. 1A). Within these data sets, the ratios of viral reads to total number of reads increased by almost 100% for captured samples, in contrast to the ratios of less than 1% for most of the unbiased NGS (Fig. 1B). The high ratio of viral to total reads in conjunction with decreased data size reduces the sequencing cost and data analysis burden. This methodology could thus greatly facilitate large-scale surveillance studies.

Once viral reads are detected in a sample, enrichment NGS can be retrospectively complemented with unbiased NGS and/or additional Sanger sequencing to obtain full-length genomes. The full-length genomes were obtained by NGS for the five cultured viruses and with minimal further gap filling for HCoV-HKU1 (240 bp), HCoV-NL63 (566 bp), and HCoV-OC43 (2,465 bp). The efficiency of CoV enrichment NGS was closely related to the number of baits and cycle threshold (*C_T_*) value (Fig. 1C and Table S2). Read depth and genome coverage were compared between unbiased NGS and enrichment NGS. The read depth for SARS-related coronavirus (SARSr-CoV), MERS-CoV, PEDV, and TGEV increased from 10- to 1,000-fold throughout the genome after enrichment. The increase in read depth can be partially attributed to the high viral titers in cultured samples. Sequencing of the full-length genome of green fluorescent protein-labeled MHV (MHV-GFP) was successful, although the read depth was lower than for unbiased NGS (Fig. 1C). An increase in read depth of at least 10-fold was observed in HCoV-NL63 and HCoV-OC43 in regions where baits were present. Sequencing of the partial genome of HCoV-HKU1 was successful with enrichment NGS, but the full-length genome was obtained with unbiased NGS (Fig. 1C). Taken together, these data indicate that enrichment NGS not only decreases the amount of data requiring analysis but can produce full-length genome coverage in both laboratory and clinical samples.

### Discovery of bat CoV genomes using capture-based NGS.

**FIG 2:**
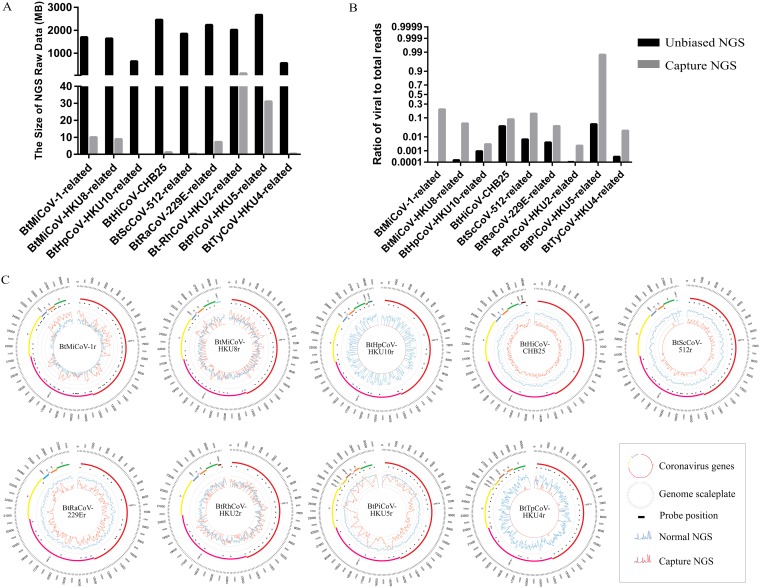
Bat CoV genome discovery using enrichment NGS. Nine bat CoV-positive samples from previous viral surveillances were used. (A) NGS data sizes were compared. (B) Ratios of viral to total reads were determined by mapping reads to the respective reference genome using CLC genomics. (C) CoV Circos plots, from outer to inner circle: CoV genome length (nt), genome annotation, CoV bait positions, read depth from direct NGS (blue lines), and read depth from enrichment NGS (red lines). Scale of read depth is shown as seven thin circular lines and ranges from 0 to 10^6^. Sample details can be found in Table S2.

The NGS pipeline was assessed for CoV discovery in bat samples. Samples from representative bat CoV species were selected based on RNA-dependent RNA polymerase (RdRp) sequence similarity to that of reference genomes. Similar to the human swab samples (Fig. 1), more data were obtained from unbiased NGS, but a higher ratio of viral to total reads was observed after enrichment (Fig. 2A and B). An increase of at least 10-fold in read depth was observed for BtMiCoV-1 (*Miniopterus bat coronavirus 1*), BtMiCoV-HKU8r (*Miniopterus bat coronavirus HKU8* related) (hereinafter, “r” denotes “related”), BtRhCoV-HKU2r (*Rhinolophus bat coronavirus HKU2* related), and BtPiCoV-HKU5r (*Pipistrellus bat coronavirus HKU5* related) in regions where baits were located (Fig. 2C). Although reads were obtained for BtRaCoV-229Er (*Human coronavirus 229E* related; sampled from *Rousettus aegyptiacus* bat) and BtScCoV-512r (*Scotophilus bat coronavirus 512* related) after enrichment, more virus-specific reads were obtained with unbiased NGS. Similarly, the efficiency of unbiased NGS was poor on BtHpCoV-HKU10r (*Bat coronavirus HKU10* related; sampled from Hipposideros pomona bat), BtHiCoV-CHB25 (related to *Bat coronavirus HKU10*; sampled from Hipposideros pomona bat), and BtTyCoV-HKU4r (*Tylonycteris bat coronavirus HKU4* related). In total, full-length genome coverage was obtained for six of nine genomes without further gap filling. More than 75% genome coverage was obtained for another 3 samples. Although complete genome coverage was obtained mostly from unbiased NGS, targeted enrichment clearly identified the presence of CoVs in bat samples. In a surveillance study, targeted enrichment is a valuable tool to triage samples for further processing.

### Diversity of bat CoV genomes.

**FIG 3:**
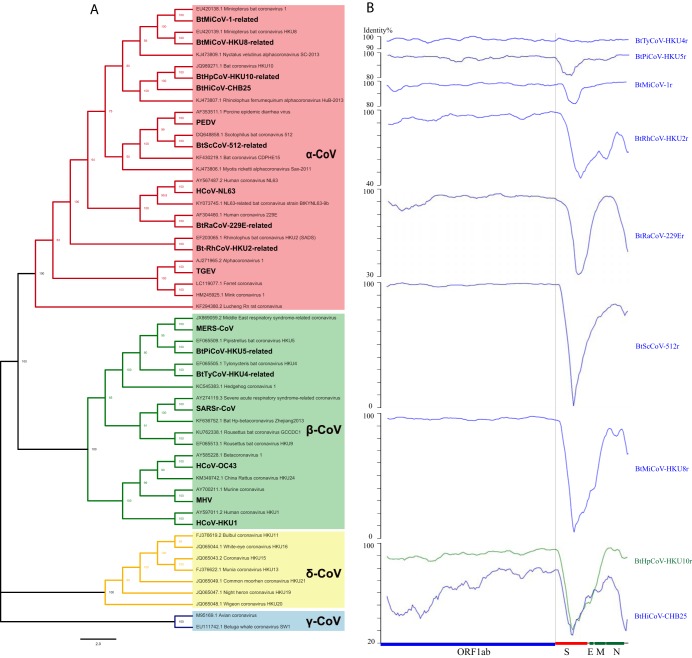
Analysis of bat CoV genomes. (A) Bayesian phylogenetic tree of ORF1b sequences from reference genomes or from CoV genomes analyzed in this study (boldface). NCBI accession numbers of reference genomes are shown. (B) Similarity plot based on the full-length genome sequences of bat CoVs. Bat CoV genomes from this study were compared to their reference genomes. The analysis was performed with the Kimura model, using a window size of 1,500 bp and a step size of 150 bp. The *x* axis illustrates typical genes in a CoV genome, and the genes are drawn to scale. The gray vertical line represents a breakpoint in most of the bat CoV species. Plots were adjusted to be the same length, as some CoVs have longer genomes. S, spike glycoprotein gene; E, small membrane protein gene; M, membrane protein gene; N, nucleocapsid protein gene. The 5'-UTR (before ORF1ab) and 3'-UTR (after N gene) regions are not shown.

To assess the diversity of nine novel bat CoV genomes, a phylogenetic tree was constructed using the conserved ORF1b protein as a reference (Fig. 3A). The newly identified viruses were most closely related to BtPiCoV-HKU5 (BtPiCoV-HKU5r), BtTyCoV-HKU4 (BtTyCoV-HKU4r), BtMiCoV-1 (BtMiCoV-1r), BtRhCoV-HKU2 (BtRhCoV-HKU2r), BtRaCoV-229E (BtRaCoV-229Er), BtScCoV-512 (BtScCoV-512r), BtMiCoV-HKU8 (BtMiCoV-HKU8r), and BtHpCoV-HKU10 (BtHpCoV-HKU10r and BtHiCoV-CHB25). In addition to the comparison with ORF1b at the protein level, the genomes of the newly identified viruses were compared to their respective reference genomes at the nucleotide level. The nucleotide sequence similarities were 97%, 96%, 96%, 89%, 85%, 91%, and 90% for BtPiCoV-HKU5r, BtTyCoV-HKU4r, BtMiCoV-1r, BtRhCoV-HKU2r, BtRaCoV-229Er, BtScCoV-512r, and BtMiCoV-HKU8r, respectively. The nucleotide sequence similarities of BtHpCoV-HKU10r and BtHiCoV-CHB25 to BtHpCoV-HKU10 were 88% and 73%, respectively (Fig. 3B).

The most divergent region of the genome was the region encoding the N terminus of the spike protein, which is usually responsible for receptor binding. In this region, seven of eight genomes showed less than 90% nucleotide identity, and five were below 40% nucleotide identity, suggesting these viruses may utilize a different receptor than their reference viruses. The most divergent, BtMiCoV-HKU8r, shared less than 10% sequence identity in this region. Another divergent region in the CoV genome is the region encoding the C terminus of the product of the N gene and the 3′ untranslated region (UTR) of the accessory protein gene (Fig. 3B). CoV accessory proteins are responsible for host response modulation and are highly variable among CoVs (1). The diversity observed in the genomes of these newly identified viruses suggests that these CoVs may be quite different in terms of receptor usage or virus-host interaction.

## DISCUSSION

Zoonotic viruses have caused most of the emerging viral disease outbreaks in recent years, and global virome surveillance programs were launched to evaluate the feasibility of preemptively mitigating pandemic threats (14). Unbiased approaches like NGS are powerful and effective, but at the same time, these methodologies are not cost-effective for routine or large-scale surveillance. Based on past experience, we expect bat CoVs to cause future outbreaks. The goal of this study was to develop an efficient and cost-effective pipeline to identify and characterize bat CoVs in future surveillance projects. Toward this end, we performed unbiased and targeted NGS on known and unknown CoVs in both laboratory and field samples.

Full-length sequences were obtained for most of the 17 CoVs in this study using unbiased NGS, but the depth of coverage differed between samples. There was an expected correlation between the amount of virus in the sample, as measured by quantitative PCR (qPCR), and the read depth obtained. PCR gap filling could be used to obtain the full-length genomes. We then compared the results of unbiased NGS with those of enrichment NGS. The CoV enrichment NGS approach with our custom bait panel resulted in good performance in most of the samples tested, but the sequencing cost was dramatically reduced. In our study, the approximate per-sample cost of HiSeq NGS (2 Gb of data) was $100, while the cost of enrichment NGS in a 10-plex sample format was approximately $60, including the hybridization and bait costs (detailed in Table S3 in the supplemental material). The cost (influenced by data size) can be further reduced by multiplexing more samples in one run. Based on the data obtained in this study, we recommend multiplexing 48 samples per sequencing run.

10.1128/mSphere.00807-19.3 TABLE S3 Cost comparison between unbiased NGS and enrichment NGS. The cost of capture NGS can be greatly reduced when dealing with multiple samples, due to the low data cost. Price is shown in Chinese yuan (CNY; 1 CNY is about $0.14 USD). Download Table S3, XLSX file, 0.03 MB. Copyright © 2020 Li et al. 2020 Li et al. This content is distributed under the terms of the Creative Commons Attribution 4.0 International license.

The use of targeted NGS for virus discovery is not new (15–17, 19). Notably, Virocap and VirCapSeq-VERT are two well-established platforms targeting viruses that infect vertebrate hosts (15, 17). Due to the broad nature of these platforms, the libraries only include a relatively small proportion of CoV baits. Furthermore, the included CoV baits are biased toward pandemic viruses, such as SARS-CoV and MERS-CoV, for which more sequence information is available in the NCBI database. The effectiveness of these baits for capturing CoVs was only tested on SARS-CoV and MERS-CoV (15, 17). We specifically designed our library to target major mammalian CoVs.

Like unbiased NGS, the depth of coverage obtained by enrichment NGS was expectedly dependent on the quantity of viral RNA in the sample. Enrichment NGS performed poorly on samples containing low viral titers. Minimal reads were obtained for the genomes of BtTyCoV-HKU4r and BtHpCoV-HKU10r, and both samples had small amounts of viral RNA (*C_T_* value of >30). The technical procedure of the capture is itself a limitation of enrichment NGS (15, 18). Key steps in the hybridization protocol, such as washes, could result in the loss of viral nucleic acid. While this loss is tolerable when the viral titer is high, a low level of viral RNA may give a false-negative result. We observed this situation with BtHpCoV-HKU10r. This virus was only detected by unbiased NGS. Similar observations have been made in previous studies, where full-length genome sequencing of human herpesvirus 1, West Nile virus, and MERS-CoV was achieved only when high viral titers were present (15, 18). We could improve the capture efficiency in two ways in the future: by using newly designed probes that bind better to their targets or by changing the steps that affect binding. For example, 65°C is the preferred temperature for Dynabeads, and thus, any step that affected the temperature would cause loss of yield. We can create a constant work condition for this step in future, or we can use different beads that require a less stringent environment. Above all, although unbiased NGS is a better choice for these samples, large-scale bat surveillance would benefit from the reduced cost of targeted enrichment. We suggest that direct NGS and gap-filling PCR are good complements to enrichment NGS once a positive sample has been identified.

While the CoV enrichment NGS successfully identified nine new CoVs, the CoV-specific enrichment also has limitations. While other enrichment NGS approaches aim to identify a broad range of known viruses across the virome (15–17), our pipeline was designed to identify known and diverse CoVs. The most challenging region to sequence was the spike gene, which has the lowest bait coverage across the genome. The genome references used in bait design do not fully reflect the diversity in this region. This is not unexpected, as this technology was not designed to detect completely novel viruses (15–17). One solution is to constantly update the baits in the CoV library to include sequence variations as they are reported (20).

Once CoVs have been identified in a sample, characterization of the full-length genome is important. Genome recombination has been documented for human CoVs, including OC43, NL63, HKU1, SARS-CoV, and MERS-CoV (2, 4). It has also been suggested that recombination between the bat SARSr-CoV strains WIV16 and Rf1-related generated a new strain, SARSr-Civet CoV SZ3, with a breakpoint at the NSP16/spike and S2 gene region (7). Breakpoints at the NSP16/spike and S2 gene region and nucleoprotein/accessory protein gene region can be found in most of the bat CoV species analyzed, suggesting that recombination is rather common. Recombination in spike or accessory proteins may generate a new virus capable of infecting via a different receptor or lead to different virus-host interactions. Genome diversity has not been assessed for CoV species like BtRhCoV-HKU2r since they were first discovered (13, 21). We should be alert and vigilant with the knowledge that bat CoVs are likely to cause another disease outbreak, not only because of their prevalence but also because the high frequency of recombination between viruses may lead to the generation of viruses with changes in virulence. BtMiCoV-HKU8r is probably a new recombinant virus that may use a different receptor than the reference virus, considering the low similarity in their spike genes. And yet, we know very little about the functionality of their accessary proteins or the biological significance of this diversity. We previously provided serological evidence that HKU8r-CoV had jumped over from bats to camels and recombined with MERS-CoV, alerting other researchers that the CoV species could be dangerous (22). Therefore, analysis of the short RNA-dependent RNA polymerase region, used in most CoV surveillance studies, is not sufficient and genome-level comparison is needed to monitor the risk of alterations in species tropism and pathogenesis.

In conclusion, we have provided a cost-effective methodology for bat CoV surveillance. The high genetic diversity observed in our newly sequenced samples suggests further work is needed to characterize these bat CoVs prior to or in the early stages of spillover to humans.

## MATERIALS AND METHODS

### Sample preparation.

Control viruses were cultured for RNA extraction. Porcine epidemic diarrhea virus (PEDV), transmissible gastroenteritis coronavirus (TGEV), MERS-CoV, SARSr-CoV, and mouse hepatitis virus (MHV) samples were cultured in Vero, swine testis (ST), Huh7, Vero E6, and DBT cells, respectively. All cells were maintained in Dulbecco modified Eagle medium (DMEM) containing 10% fetal bovine serum (FBS) and incubated at 37°C with 5% CO_2_. Once cytopathic effect (CPE) was observed, 140 μl of supernatant was collected for RNA extraction.

To analyze clinical samples, HCoV-NL63, HCoV-OC43, and HCoV-HKU1 were extracted from human oral swabs. RNA for BtMiCoV-1r, BtMiCoV-HKU8r, BtHpCoV-HKU10r, BtHpCoV-CHB25, BtScCoV-512r, BtRaCoV-229Er, BtRhCoV-HKU2r, BtPiCoV-HKU5r, and BtTyCoV-HKU4r, which were collected during previous bat CoV surveillance projects, was extracted from bat rectal swabs (11, 23, 24). To process RNA, 560 μl of buffer AVL (Qiagen) was added to the tube containing 140 μl swab sample or culture supernatant. Samples were vortexed for 15 s and then centrifuged at 12,000 × *g* for 10 min to obtain a clear supernatant. Viral nucleic acid was extracted using the QIAamp viral RNA minikit (Qiagen) following the manufacturer’s instructions.

### qPCR.

For quantitative PCR (qPCR) analysis, primers based on the CoV *RdRp* gene were used (Table S1 in the supplemental material). RNA was reverse transcribed using PrimeScript RT master mix (TaKaRa). The 10-μl qPCR mixture contained 5 μl 2× SYBR premix Ex Taq II (TaKaRa), 0.4 μM each primer, and 1 μl cDNA. Amplification was performed as follows: 95°C for 30 s, followed by 40 cycles at 95°C for 5 s and 60°C for 30 s and an additional melt step.

10.1128/mSphere.00807-19.1 TABLE S1 qPCR primer sequences for CoVs. Download Table S1, XLSX file, 0.1 MB. Copyright © 2020 Li et al. 2020 Li et al. This content is distributed under the terms of the Creative Commons Attribution 4.0 International license.

### Preparation of Illumina DNA libraries from RNA.

Libraries for NGS were constructed from total RNA using the TruSeq stranded mRNA library preparation kit for Illumina (Illumina) according to the manufacturer’s instructions. Briefly, 8 μl of total RNA was added to first-strand synthesis buffer and random primers before a 4-min incubation at 94°C to generate RNA fragments larger than 300 nucleotides (nt). Following first- and second-strand cDNA synthesis, double-stranded cDNA was purified using Agencourt AMPure XP beads (Beckman Coulter Genomics) and eluted in 20 μl nuclease-free H_2_O. To obtain a library size larger than 300 nt, the library was amplified by PCR using the following conditions: initial denaturation at 98°C for 30 s, 10 cycles of denaturation for 10 s at 98°C, annealing for 30 s at 60°C, and extension for 30 s at 72°C, and then a final extension for 5 min at 72°C. Libraries were purified using Agencourt AMPure XP beads (Beckman Coulter Genomics), eluted in 10 μl nuclease-free H_2_O, visualized on a 1.5% agarose gel, and quantified using a Bioanalyzer high-sensitivity DNA assay (Agilent). Once prepared, the libraries were divided in two. Half the library was sequenced directly to obtain the unbiased reads, and half was enriched prior to NGS.

### Enrichment of CoV sequences in libraries.

Targeted CoV genome enrichment was achieved using our customized biotinylated 120-mer xGen Lockdown baits (Integrated DNA Technologies) (18). Prior to capture of viral sequences, 2 μl of xGen universal blocker-TS mixture (Integrated DNA Technologies), matched according to the library index, was added to 20 μl of library DNA. To block binding of baits to nonviral regions of library fragments, 0.5 μl of 5 μg Cot-1 DNA (Invitrogen) was added. Blocked libraries were ethanol precipitated and resuspended in 2.5 μl of nuclease-free H_2_O, 3 μl NimbleGen hybridization solution, and 7.5 μl NimbleGen 2× hybridization buffer (Roche). Following a 10-min incubation at room temperature, resuspended libraries were denatured at 95°C for 10 min and cooled on ice before the addition of the CoV bait pool. A total of 3 pmol of baits was added and hybridized to the libraries for 4 h at 65°C. To capture virus-specific library fragments, 100 μl of Dynabeads M-270 streptavidin magnetic beads (Life Technologies) was added to the hybridization reaction mixture and the mixture was incubated for a further 45 min at 65°C with shaking at 2,000 rpm on a ThermoMixer C shaker (Eppendorf). Streptavidin beads were washed to remove unbound DNA, using the SeqCap EZ hybridization and wash kit (Roche) according to the manufacturer’s instructions. A postcapture PCR amplification with P1 and P2 primers (Illumina) was performed using the following conditions: initial denaturation at 95°C for 2 min, 20 cycles of denaturation for 20 s at 95°C, annealing for 20 s at 65°C, and extension for 15 s at 72°C, and then a final extension step for 3 min at 72°C. The enriched library was purified using Agencourt AMPure XP beads (Beckman Coulter Genomics) and eluted in 10 μl nuclease-free H_2_O, visualized on a 1.5% agarose gel, and quantified using a Bioanalyzer high-sensitivity DNA assay (Agilent). All samples were subjected to the same library preparation and enrichment.

### Data analysis.

Each unbiased NGS library was run on one HiSeq lane. The 17 enriched libraries were made into two pools (8 or 9 per each) and run on HiSeq lanes. NGS reads were assembled into genomes using the Galaxy platform (25). PCR and Sanger sequencing were used to fill the genome gaps. All genomes were interrogated for ORFs using ORFfinder (https://www.ncbi.nlm.nih.gov/orffinder/). The search parameters were set to ignore nested ORFs and filter out ORFs of less than 150 bp. The standard genetic code and the “ATG only” rule were selected. Each ORF was identified and annotated through BLASTN and BLASTX using the NCBI database. Read mapping or PCR resequencing was used to verify novel ORFs. Read depth was assessed by mapping reads from direct or enriched NGS to their respective genomes using CLC Genomics Workbench version 12.0 (Qiagen). Bait positions were calculated by aligning baits to each genome by BLASTN. The ratio of viral reads to total reads was calculated for each sample. The ORF1b sequences of 38 ICTV reference genomes and 17 CoV genomes from this study were aligned by ClustalW (version 2.1). The phylogenetic tree was generated using the neighbor-joining method in the maximum-composite-likelihood model in MEGA (version 7.0.18) with nucleotide substitution type and 1,000 bootstrap iterations. The schematic diagrams of CoV genomes, including bait positions and read depths of NGS, were prepared using Circos (version 0.69.8). Graphs displaying the data size and viral read ratios were generated using Prism (GraphPad Prism 7).

### Data availability.

Viral genome data for new CoVs obtained from this study are available in GenBank under accession numbers MN611517 to MN611525.
