# Correction for Li et al., “Discovery of Bat Coronaviruses through Surveillance and Probe Capture-Based Next-Generation Sequencing”

**DOI:** 10.1128/mSphere.00170-20

**Published:** 2020-03-18

**Authors:** Bei Li, Hao-Rui Si, Yan Zhu, Xing-Lou Yang, Danielle E. Anderson, Zheng-Li Shi, Lin-Fa Wang, Peng Zhou

**Affiliations:** a CAS Key Laboratory of Special Pathogens, Wuhan Institute of Virology, Center for Biosafety Mega-Science, Chinese Academy of Sciences, Wuhan, China; b University of Chinese Academy of Sciences, Beijing, China; c Programme in Emerging Infectious Diseases, Duke-NUS Medical School, Singapore, Singapore

## AUTHOR CORRECTION

Volume 5, no. 1, e00807-19, 2020, https://doi.org/10.1128/mSphere.00807-19. Page 9: the following sentence should be added to the “Data availability” paragraph: “Raw next-generation sequencing data have been deposited in the NCBI SRA database under accession no. SRR11085733 to SRR11085741.”

